# Dual conception of risk in the Iowa Gambling Task: effects of sleep deprivation and test-retest gap

**DOI:** 10.3389/fpsyg.2013.00628

**Published:** 2013-09-19

**Authors:** Varsha Singh

**Affiliations:** Humanities and Social Science, Indian Institute of Technology DelhiNew Delhi, India

**Keywords:** Iowa Gambling Task, decision making, risk, reward–punishment, sleep deprivation, test-retest gap

## Abstract

Risk in the Iowa Gambling Task (IGT) is often understood in terms of intertemporal choices, i.e., preference for immediate outcomes in favor of delayed outcomes is considered risky decision making. According to behavioral economics, healthy decision makers are expected to refrain from choosing the short-sighted immediate gain because, over time (10 trials of the IGT), the immediate gains result in a long term loss (net loss). Instead decision makers are expected to maximize their gains by choosing options that, over time (10 trials), result in delayed or long term gains (net gain). However, task choices are sometimes made on the basis of the frequency of reward and punishment such that frequent rewards/infrequent punishments are favored over infrequent rewards/frequent punishments. The presence of these two attributes (intertemporality and frequency of reward) in IGT decision making may correspond to the emotion-cognition dichotomy and reflect a dual conception of risk. Decision making on the basis of the two attributes was tested under two conditions: delay in retest and sleep deprivation. An interaction between sleep deprivation and time delay was expected to attenuate the difference between the two attributes. Participants were 40 male university students. Analysis of the effects of IGT attribute type (intertemporal vs. frequency of reinforcement), sleep deprivation (sleep deprivation vs. no sleep deprivation), and test-retest gap (short vs. long delay) showed a significant within-subjects effect of IGT attribute type thus confirming the difference between the two attributes. Sleep deprivation had no effect on the attributes, but test-retest gap and the three-way interaction between attribute type, test-retest gap, and sleep deprivation were significantly different. *Post-hoc* tests revealed that sleep deprivation and short test-retest gap attenuated the difference between the two attributes. Furthermore, the results showed an expected trend of increase in intertemporal decision making at retest suggesting that intertemporal decision making benefited from repeated task exposure. The present findings add to understanding of the emotion-cognition dichotomy. Further, they show an important time-dependent effect of a universally experienced constraint (sleep deprivation) on decision making. It is concluded that risky decision making in the IGT is contingent on the attribute under consideration and is affected by factors such as time elapsed and constraint experienced before the retest.

## Introduction

The Iowa Gambling Task (IGT; Bechara et al., 1994) is used to test a hypothesis about emotion and decision making called the somatic marker hypothesis (SMH; Damasio, 1994). The main assumption in the SMH–IGT framework is that risk is perceived in terms of its intertemporal attribute, i.e., choice of immediate as opposed to delayed reward and punishment is considered risky (Bechara et al., 2005). However, IGT task choices also differ on the basis of the frequency of immediate rewards and punishments; thus, task choices differ in two ways. To clarify, the IGT offers a choice among four decks of cards, labeled A′, B′, C′, and D′. Unlike the original paper-and-pencil based task (ABCD), the computerized task (A′B′C′D′) has increased delayed punishment and therefore it amplifies the effect of disadvantageous choices (see Bechara et al., 2000 for differences between the two variants). Unbeknown to the decision maker, decks A′ and B′ have high immediate rewards (100 points per card-pick) with 50% of cards drawn from deck A′ giving a loss of 35–100 points and 10% of cards drawn from deck B′ giving a loss of 1250 points, such that 10 cards drawn from decks A′ and B′ result in a net loss of 250 points. Decks C′ and D′ have small immediate rewards (50 points per card-pick) with 50% of cards drawn from deck C′ giving a loss of 25–75 points and 10% of cards drawn from deck D′ giving a loss of 250 points, such that 10 cards drawn from decks C′ and D′ result in a net gain of 250 points. Therefore the four decks differ in two ways: (a) net outcome across time (i.e., inter temporal attribute) by which decks A′ and B′ could be considered risky in the long term, whereas decks C′ and D′ could be considered safe in the long term, and (b) frequency of immediate reward and punishment notwithstanding net or long-term outcomes (i.e., frequency attribute) by which decks A′ and C′ could be perceived as risky due to frequent punishments/infrequent rewards and decks B′ and D′ could be perceived as safe due to infrequent punishments/frequent rewards.

It is commonly understood that risk perception and decision making in the IGT is governed by the intertemporal attribute (Bechara et al., 2005), and that choices on the basis of the frequency attribute have no long-term advantage (Dunn et al., 2006). Nevertheless, there have been many observations of decision making on the basis of the frequency attribute (Wilder et al., 1998; Ritter et al., 2004; Bark et al., 2005; Fishbein et al., 2005; Shurman et al., 2005; Toplak et al., 2005; van den Bos et al., 2006). This preference is incompatible with the SMH–IGT framework as demonstrated, for example, by the finding that deck B′ was considered “risky” on the basis of the intertemporal attribute and is preferred to other “safe” decks (Lin et al., 2007), whereas deck C′ that was considered “safe” is avoided by healthy participants (Chiu and Lin, 2007). Furthermore, dispositional risk seekers who were assessed using a modified risk-taking scale (Domain-Specific Risk-Taking; Weber et al., 2002) preferred decks A′ and C′ and avoided decks B′ and D′ (Singh and Khan, 2008). Together, these findings suggest that, in the IGT, risk might be perceived in two ways, either by the intertemporal attribute or by frequency of reward and punishment.

This dual conception of risk in the form of two attributes (intertemporality and frequency) represents an important dichotomy of cognition-emotion in IGT decision making. Support for this dichotomy comes from dual process theories of reasoning according to which there are two systems that process information differently. One system is automatic, emotion-based, and concerned with the present, whereas the second is reflective, cognition-based, and concerned with the future (Tversky and Kahneman, 1971). Decision making on the basis of the intertemporal attribute in the IGT reflects explicit learning (Maia and McClelland, 2004), is dependent on hippocampus-mediated memory systems such as the declarative memory system (Gupta et al., 2009), engages working memory (Hinson et al., 2002), and requires cognitive processing (Stocco et al., 2009). However, decision making on the basis of the frequency attribute is attributed to automatic processing (Wilder et al., 1998; Stocco et al., 2009). These findings suggest that decision making on the basis of the intertemporal attribute is the result of activity in the cognition-based system whereas decision making on the basis of the frequency attribute may reflect activity in the emotion-based system. Indeed, Stocco et al. (2009) found a double dissociation in decision making on the basis of the two attributes (intertemporality and frequency). These researchers tested the role of cognitive resources first by introducing a secondary task during learning of the deck payoffs, and second, by restricting display of the outcome, that is, by restricting access to information about the deck payoffs. Contrary to their expectation, absence of a secondary task (working memory load) was associated with greater decision making on the basis of the frequency attribute. Thus, absence of a secondary task, assumed to benefit the cognition-based system, instead appeared to benefit the emotion-based system.

Unlike previous research, the present study was aimed at differentiating decision making on the basis of the two attributes (intertemporality and frequency) by manipulating re-test gap and sleep deprivation, factors known to influence decision making on the IGT. The dual process theory suggests that task-familiarity (e.g., at retest) is conducive to activity of cognition-based system (rather than to activity of emotion-based system). Accordingly, decision making on the basis of the intertemporal attribute is observed to improve at retest (i.e., preference for safe long-term advantageous decks increases at retest) (Bechara et al., 2000); this supports the contention that intertemporal decision making is cognition-based. However, it is unclear whether task-familiarity at the retest reduces the reliance on emotion-based system and results in a decrease in decision making on the basis of the frequency attribute (i.e., preference for infrequent punishment—frequent rewards decks decreases at retest).

Furthermore, the difference between the two attributes should be attenuated by two factors: (1) time delay, i.e., test-retest gap, and (2) sleep deprivation. For example, it has been observed that a lengthy (1 month) test-retest gap strengthens intertemporal decision making much more (i.e., greater increase in choices made from the long-term advantageous decks) (Bechara et al., 2000) than a shorter (1 week) test-retest gap (Turnbull and Evans, 2006) suggesting that task familiarity offered by a retest and a long test-retest gap and benefits intertemporal attribute. The present study investigates the interaction between attribute type and the test-retest gap. Few studies have investigated the effects of sleep deprivation on the IGT (e.g., Killgore et al., 2006, 2007), however none have compared decision making on the basis of both the attributes. Sleep deprivation impairs performance on tasks that rely on the explicit memory system (Drosopoulos et al., 2005; Fischer et al., 2006), it is the same system that governs intertemporal decision making in the IGT (Maia and McClelland, 2004). Although decision making is often analyzed only on the basis of intertemporality (i.e., the cognition-based system) (e.g., Killgore et al., 2006, 2007), the impairment caused by sleep deprivation has been explained as a failure of integration of both cognitive and affective processes (Killgore et al., 2007). This makes it essential to understand the effects of sleep deprivation on both attributes of decision making in the IGT.

A few studies have investigated the combined effects of sleep deprivation and test-retest gap on the IGT (e.g., Killgore et al., 2006, 2007) however, none have compared decision making on the basis of both attributes. Killgore et al. (2006) found that a short (1 day) test-retest gap, when combined with sleep deprivation, impaired decision making and increased risky choices in the IGT (greater number of choices made from the short-term advantageous decks). At least one animal study has shown that sleep deprivation and a short test-retest gap disrupt learning of a hippocampus-dependent task, whereas sleep deprivation fails to cause a disruption with a longer delay (Graves et al., 2003) suggesting that the effects of sleep deprivation might be time-dependent. As pointed out earlier, intertemporal decision making is dependent on hippocampus-mediated memory systems (Gupta et al., 2009), therefore, it was expected that a long test-retest gap would reduce sleep deprivation impairments on the IGT in the case of the intertemporal attribute. Overall, for the intertemporal attribute, a short test-retest gap and sleep deprivation is expected to inhibit performance whereas a long test-retest gap is expected to counteract (at least partially) the negative effects of sleep deprivation. Thus, the present study was focused on the interaction between sleep deprivation and test-retest gap and it was expected that this interaction would attenuate the difference between the two attributes (intertemporality and frequency).

The research aims of the present study were to compare the two attributes (intertemporality and frequency of immediate reinforcement) when conditions were varied along two dimensions, test-retest gap and sleep deprivation. It was hypothesized that decision making would differ across the type of attribute (intertemporal/frequency of reinforcement); it was expected that sleep deprivation (sleep deprived/not sleep deprived) and test-retest gap (short/long delay) would affect the two attributes differently. A three-way interaction between attribute type, sleep deprivation condition, and test-retest gap was expected. Specifically, advantageous intertemporal decision making (i.e., net scores calculated on the basis of intertemporal attribute) was expected to decrease under conditions of sleep deprivation and short test-retest gap.

## Materials and methods

### Sample

Forty healthy, non-smoking, right-handed, Indian male students volunteered for the study (Mean age = 24.92 years; *SD* = 1.99). Even though the use of caffeine does not reverse sleep deprivation impairments in intertemporal decision making (Killgore et al., 2007), self-reported consumption of tea/coffee greater than 4–5 cups per day was an exclusion criterion. An all-male sample was employed because gender plays a critical role in sleep-deprivation-related risk behavior (Acheson et al., 2007) and in IGT decision making (Tranel et al., 2005). In addition, female students were reluctant to stay overnight (a condition of testing) due to the sociocultural environment (gender roles) of the country where the research was conducted (i.e., India). All participating students were enrolled in a PhD program in either the Department of Biosciences and Bioengineering (90%) or the Department of Humanities and Social Sciences (10%). The students were told that the study aimed to understand decision making and would require them to be available for two sessions. No incentives (money or course credit) were offered because these could produce each participant's superficially “best” task performance rather than mimic real or natural task performance.

### Design and analysis

A 2 × 2 × 2 mixed repeated-measures design was employed with scoring type (intertemporal; frequency of reinforcement), sleep deprivation (sleep deprived; not sleep deprived), and test-retest gap (short; long) as factors. The analysis was repeated on the first factor. The variables were the difference between total net IGT scores at retest (T2) and baseline (T1) sessions, (1) scored according to the intertemporal attribute [T2 ((C′ + D′) − (A′ + B′)) − T1 ((C′ + D′) − (A′ + B′))] and (2) scored according to frequency of preference for immediate reinforcement [T2((B′ + D′) − (A′ + C′)) – T1((B′ + D′) − (A′ + C′))].

In the present study, the difference between total net IGT scores at test (T1) and retest (T2) is considered. This differs from previous studies where decision making was analyzed using block-wise scores at retest (T2). Such studies have either used an alternate version of the IGT at retest (Killgore et al., 2006), or have changed the deck payoffs at retest (Turnbull and Evans, 2006) to maintain uncertainty in decision making at retest. This method of analyzing block-wise performance is appropriate for comparing participants' rates of learning across trials because the initial trials of the IGT (even at baseline) are considered to involve decision making under uncertainty, whereas latter trials are considered to involve decision making under risk or known payoffs (Brand et al., 2007). However, the present study aimed to test decision making under risk (i.e., under knowledge of payoffs) rather than under uncertainty (i.e., under none/partial knowledge of payoffs). Therefore, it was deemed acceptable to utilize a consistent variant of the IGT with the same deck payoffs throughout the entire study. Furthermore, in a sleep deprivation study, Killgore et al. (2006) used a within-subjects design, that is, participants served as their own controls, a design that did not require accounting for differences between the participants at the baseline, making it appropriate to analyze decision making only at retest. However, the present study used a mixed design therefore it was essential to take into account differences in performance at baseline (T1) and retest (T2) for all participants.

### Materials

A computerized version of the IGT (A′B′C′D′) and task instructions were presented on a computer screen. There were 60 cards in a deck, and the exclusion criterion was exhausting any of the four decks at either Time 1 (T1) or at Time 2 (T2); none of the participants exhausted a deck. In the present study, deck pay offs matched those used by Bechara et al. (2000) such that the task amplified the negative consequences of selecting disadvantageous decks.

### Procedure

Participants filled in a questionnaire giving their demographic information. They were then presented with an overview of the experiment, and gave informed consent. Participants were also informed that their participation was voluntary, and that they could drop out of the experiment at any stage. The study received the approval of three committees comprising interdisciplinary experts: (1) a thesis committee (Research Progress Committee), (2) a departmental committee, and (3) an institute-level committee for the post-graduate research program (competent authority for giving clearance for conducting research on human participants). Participants were then randomly assigned to one of four groups (short-test-retest gap/sleep deprivation, long test-retest gap/sleep deprivation, short test-retest gap/no sleep deprivation, or long test-re-test delay/no sleep deprivation). Each participant was tested to measure baseline IGT decision making (T1 consisted of 100 trials). The two groups with short test-retest gaps were retested (T2 consisted of 100 trials) 24 h after the baseline session. However, the two groups with long test-retest gap were retested 12 weeks after the baseline session. Participants in the sleep-deprivation conditions were retested after one night of sleep deprivation and participants in the no-sleep-deprivation conditions were retested after a single restful night of sleep. Sleep deprivation introduced immediately after baseline (T1) and a long test-retest gap would have allowed investigation of post-task learning and consolidation; however the focus of the present experiment was on comparing decision making on the basis of the two attributes (intertemporality and frequency) based on the presence (or absence) of sleep deprivation and the length of test-retest gap.

All participants spent the night before the retest session in a dormitory in the presence of a male research assistant who observed no tossing and turning or other discomfort among the participants as they slept. The environment matched that of dormitories that are a regular feature of student life in the engineering institutes in India. The dormitory had furniture (beds, tables, chairs, side-tables, ceiling fans), lighting (tube lights), and computers, and was maintained at a temperature similar to the students' own rooms. Participants in the sleep-deprivation group were allowed to read books or magazines, watch movies, or complete college assignments while in the dormitory room. Participants in the sleep-deprivation group were in the company of a male research assistant and refrained from drinking caffeinated beverages (e.g., tea, coffee) throughout the night. Participants in the no–sleep-deprivation group were asked to sleep (in presence of a male research assistant who observed no discomfort among participants, and who woke participants in time for the retest session). All participants were discouraged from taking afternoon naps the day before the retest and were reminded not to discuss the study with others. All IGT testing was done between 7:00 a.m. and 9:00 a.m., and in each retest half of participants were sleep-deprived and half were not. Baseline and retest times were matched for all participants; for example, if a participant underwent the baseline session at 8.00 a.m., his or her retest session was also at 8.00 a.m.

## Results

Table 1 gives descriptive statistics for the IGT decision-making scores, calculated in two ways (intertemporality and frequency) for the four groups. Larger standard deviations (suggesting greater variability) have been observed for intertemporal decision making in the IGT (Bowman and Turnbull, 2003; Newman et al., 2008) and were also observed in the present study.

**Table 1:** **Groupwise differences between total net IGT scores at retest and baseline (T2 – T1), calculated according to the intertemporality and frequency attributes (*n* = 40)**.

**Scoring/attribute type**	**Groups**			
	**Long time/Sleep dep.**	**Short time/Sleep dep.**	**Long time/No sleep dep.**	**Short time/No sleep dep.**
Intertemporal attribute [(C′ + D′) − (A′ + B′)]	28.30 (40.22)	−17.40 (17.61)	20.50 (27.83)	10.60 (19.10)
Frequency of reinforcement attribute [(B′ + D′) − (A′ + C′)]	−14.80 (21.99)	−04.80 (19.65)	01.70 (35.13)	−07.80 (20.94)

Note: Values given are means (standard deviations). Long time/Sleep dep., Long test-retest gap, sleep deprivation; Short time/Sleep dep., Short test-retest gap, sleep deprivation; Long time/No sleep dep., Long test-retest gap, no sleep deprivation; Short time/No sleep dep., Short test-retest gap, no sleep deprivation.

There was a significant main effect of attribute type, *F*_(1, 36)_ = 7.51, *p* < 0.01, η^2^_*p*_ = 0.17. The was a significant interaction effect between attribute type and test-retest gap, *F*_(1, 36)_ = 5.01, *p* < 0.05, η^2^_*p*_ = 0.12. There was also a significant three-way interaction among attribute type, sleep deprivation, and test-retest gap, *F*_(1, 36)_ = 5.16, *p* < 0.05, η^2^_*p*_ = 0.12. The results showed a difference between the total net scores calculated on the basis of two different conceptualizations of risk in IGT—one based on the intertemporal nature of reward and punishment and the other based on preference for a specific frequency of immediate reward and punishment. The test-retest gap interacted with attribute type suggesting that risk taking (as understood according to the two different attributes) is differentially susceptible to time delay between the two exposures to the IGT. Contrary to expectations, sleep deprivation did not have an effect on IGT decision making analyzed via the two attributes. However, the three-way interaction between sleep deprivation, time delay, and attribute type was significant.

**Figure 1:**
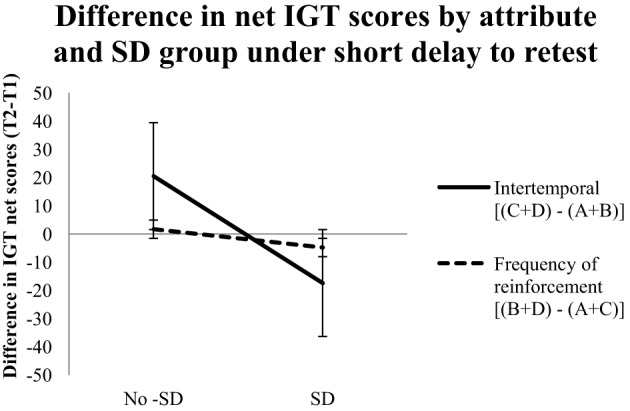
**IGT scores for a short (24 h) test-retest gap.** Mean difference between retest (T2) and baseline (T1) total net IGT scores (100 trials per session) analyzed via the two attributes (intertemporal attribute and frequency of reinforcement attribute) for the no-sleep-deprivation (No-SD) and sleep-deprivation (SD) conditions. Error bars indicate standard error of the mean.

**Figure 2:**
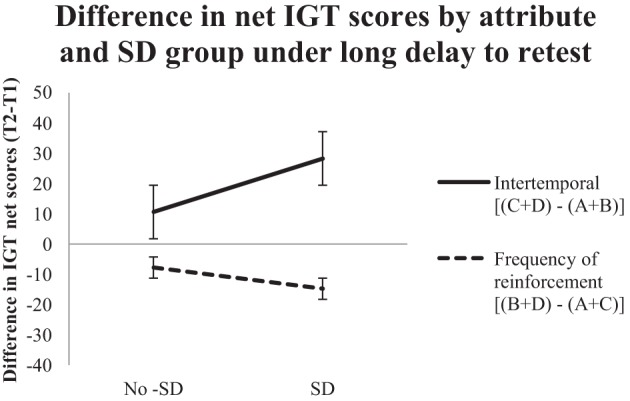
**IGT scores for a long (12 weeks) test-retest gap.** Mean difference between retest (T2) and baseline (T1) total net IGT scores (100 trials per session) analyzed via the two attributes (intertemporal attribute and frequency of reinforcement attribute) for the no-sleep-deprivation (No-SD) and sleep-deprivation (SD) conditions. Error bars indicate standard error of the mean.

To further investigate the role of the test-retest gap, the three-way interaction (attribute type × time delay × sleep deprivation) was further probed with a repeated measures ANOVA on the data that was split according to the short and long test-retest gaps. For the short test-retest gap, the effect of attribute type was not significant, but the interaction between sleep deprivation and attribute type was significant, *F*_(1, 18)_ = 4.55, *p* < 0.05, η^2^_*p*_ = 0.20. In contrast, for the long test-retest gap, there was a significant effect of attribute type, *F*_(1, 18)_ = 9.61, *p* < 0.01, η^2^_*p*_ = 0.35, whereas the interaction of sleep deprivation with attribute type was not significant. These results suggest that the difference between the two attributes is unaffected by a short test-retest gap, but that sleep deprivation introduced with a short test-retest gap attenuates the difference between the two attributes. Conversely, the difference between the two attributes is affected by a long test-retest gap, but the difference between the two attributes is unaffected by introducing sleep deprivation with a long test-retest gap. Figures 1, 2 depict the time-dependent effects (short vs. long test-retest gap, respectively) of sleep deprivation on the two attributes.

**Figure 3:**
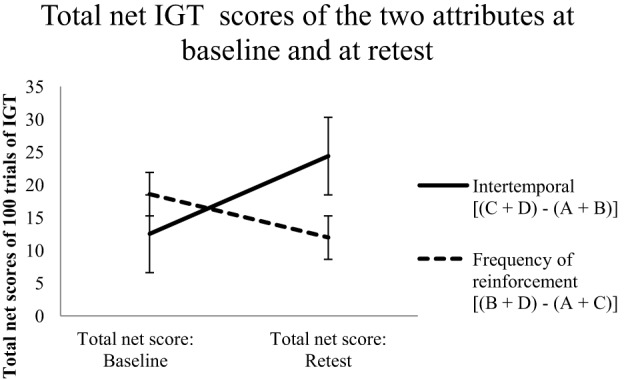
**Total net IGT scores at baseline and retest calculated using two methods of scoring risky decision making.** The intertemporal attribute shows a strengthening of preference for delayed outcomes at retest whereas the frequency attribute does not. Error bars indicate standard error of the mean.

To test whether there was a difference between decision making at retest (T2) and at baseline (T1) for the two attributes (i.e., to test whether decision making at T2 was different from that at T1), a paired *t*-test was done for total net scores derived via the two attributes. There was a significant improvement in total net IGT scores between baseline (*M* = 12.53, *SD* = 33.05) and retest (*M* = 24.38, *SD* = 24.09) when scored on the basis of intertemporal attribute [i.e., (C + D) – (A + C)], *t*_(39)_ = −2.32, *p* < 0.05. However, when total net IGT scores were calculated according to preference for immediate reinforcement, they showed a slight decline from baseline (*M* = 18.58, *SD* = 19.38) to retest (*M* = 11.95, *SD* = 26.12); however, this difference was not significant (Figure 3). As expected, the results suggested that, overall, performance on the basis of the intertemporal attribute increased with an increase in task exposure.

## Discussion

The present study tested dual conception of risk in the IGT as manifested by two decision making attributes (intertemporal attribute and frequency of reward and punishment). As expected, The ANOVA showed a difference between the two total net IGT scores derived from the two attribute types (intertemporality and frequency). Thus, the data support the hypothesis that there is a distinction between the two conceptualizations of risk in the IGT indicating that decision making of cognition-based system differs from that of emotion-based system at the retest.

As expected, differences in the two attributes were affected by the length of test-retest gap suggesting that temporal stability in risk taking is contingent both on the attribute under consideration and on the time gap between test and retest. Contrary to expectations, sleep deprivation had no independent effect on the two attributes. This could be, in part, because rewards and punishments are present in both attribute types, and because sleep deprivation alters risk differentially for reward and punishment (McKenna et al., 2007; Venkatraman et al., 2007). For instance, decision making on the basis of frequency of reinforcement (i.e., cards drawn from decks B′ and D′) is thought to reflect a preference for frequent rewards rather than for infrequent punishments (Wilder et al., 1998; Lin et al., 2007). Decision making in the IGT is believed to be complex in nature IGT (Upton et al., 2011). For instance, when sleep deprivation induces risk-taking (intertemporal risk) and such risk-taking is tested via the IGT, the mitigating effects of caffeine cannot be observed (Killgore et al., 2007). However, when risk-taking was tested via another task, called the Ballon Analog Risk Task (BART), caffeine was found to restore risk taking (in sleep-deprived individuals) to baseline level (Killgore et al., 2008). Killgore et al. attribute this difference in the mitigating effects of stimulants to the fact that the IGT has a “gain” frame whereas the BART has a “loss” frame Killgore et al. (2008). In fact, it is believed that risk perception in the IGT may further differ between the domains of reward and punishment (Levin et al., 2012).

In support of a hypothesized distinction between the attributes, sleep deprivation in conjunction with the test-retest gap had a significant effect on the two attributes. This suggests that the length of a test-retest gap plays a crucial role in how sleep deprivation affects risky decision making when conceptualized in two different ways. Follow-up analysis showed that a short test-retest gap did not affect the two attributes differentially, but that introducing sleep deprivation with a short test-retest gap enhanced the difference between the two attributes. On the other hand, a long test-retest gap did affect the two attributes differentially and introducing sleep deprivation after a long delay did not have any differential effect on the two attributes. These results are consistent with those of Killgore et al. (2006, 2007) in which sleep deprivation and short test-retest gaps (49, 51, 72 h) impaired intertemporal decision making. It is possible that the combination of a short test-retest gap and sleep deprivation creates fatigue which promotes dichotomizing of the two attributes. This explanation is aligned with that given by Killgore et al., in which fatigue due to sleep deprivation Killgore et al. (2007) or due to even a modest self-reported decrease in sleep duration Killgore et al. (2012) is believed to contribute to a failure of cognition-emotion integration. In other words, fatigue might contribute to differentiation of cognition and the emotion-based system. Even though the present study did not test post-task consolidation, it is possible that the effect of sleep deprivation is time dependent for the widely used intertemporal attribute in the IGT. The current results imply that temporal stability of the two attributes is different and that learning of the two attributes might be differentially vulnerable to the effects of test-retest gap and sleep deprivation.

As expected, repeated task exposure (by retest) appeared to be conducive to activity of cognition-based system. At retest, there was a marked increase in choices made on the basis of the intertemporal attribute. In the IGT, the intertemporal attribute embodies a common conception of risk; that is, risk is considered as an anticipated tradeoff between immediate and delayed outcomes. On the other hand, choices made on the basis of the frequency-of-reinforcement attribute suggest that risk perception in the IGT may be automatic and reflect spontaneous processing of the frequency of rewards and punishments (Stocco et al., 2009). In line with dual process theory (e.g., Evans, 2003; Kahneman and Frederick, 2007), the frequency attribute may be the “default attribute” and decision making on the basis of the intertemporal attribute may require inhibition or overriding of this “default” mode. For example, in the present study, it is possible that repeated task exposure at retest overrode the response of the emotion-based system while at the same time strengthening intertemporal decision making. This dual conception of risk in the IGT is aligned with the behavioral decision-making literature that considers risk as “anticipated as well as anticipatory” and “a deliberate as well as instinctive process” (Loewenstein et al., 2001; Slovic et al., 2004, 2005; Slovic and Peters, 2006).

Consistent with the findings of Kahneman and Frederick (2007), the present results indicate that two distinct types of reasoning and rationality are manifested in IGT decision making. Could the conceptualization of risk and rationality advanced by the SMH–IGT framework—that is, risk as an intertemporal choice and rationality as making long term advantageous decisions—be a reflection of the environment where the task was developed and the cognitive demands of that environment? Decision making in the IGT is observed to be governed by frequency of reinforcement rather than the intertemporal attribute in several cultural contexts including Taiwan (Chiu and Lin, 2007; Lin et al., 2007; Chiu et al., 2008), Iran (Ekhtiari et al., 2009), Brazil (Schneider et al., 2010), and India (Singh and Khan, 2008). Future studies could utilize the IGT to understand cultural variations in risk perception and risk taking at the behavioral as well as the neural level.

Apart from the small sample size and the use of an all-male sample, the present study had other limitations, such as the lack of physiological monitoring to ascertain the effects of sleep deprivation and a lack of accounting for individual disposition (Franken and Muris, 2005) and mood (Suhr and Tsanadis, 2007). One disadvantage of varying the test-retest gap (short and long test-retest gap) is the inability to equate the affective and motivational states of the two groups between the two testing sessions. Importantly, studying the effects of both, sleep and test-retest gap on the IGT decision-making task will require weighing the advantages and disadvantages of the research paradigm utilized (Pace-Schott et al., 2011). For example decision making without reward (as in the present IGT study) has the advantage of ensuring that performance does not depend on incentives that compensate for the effects of sleep deprivation and that performance is not bolstered by reward incentives; a lack of incentive can be disadvantageous in that it may be difficult to make inferences about motivation in a decision making task where no incentive is provided. However, at least one study has shown that there is no difference in IGT decision making based on whether incentives are real (monetary) or facsimiles (Bowman and Turnbull, 2003).

## Conclusion

The present results contribute to current understanding of IGT decision making related to two important attributes, the intertemporal attribute and the frequency of reinforcement attribute. The results also add to knowledge concerning the larger question of the dichotomy between cognition and emotion in decision making. For instance it might be possible that failure to incorporate the cognition and emotion dichotomy is responsible for the instability that is observed in risky decision making (Fox and Tannenbaum, 2011; Vlaev, 2011). Apart from pointing out that stability in risk taking in the IGT is contingent on the attribute under consideration, the current results also suggest that inconsistency in risk taking (across a time span) observed in decision making tasks could be due to factors (such as time elapsed and constraint) affecting dichotomization of the emotion-cognition processes.

### Conflict of interest statement

The author declares that the research was conducted in the absence of any commercial or financial relationships that could be construed as a potential conflict of interest.
